# Supplementary material for: Chemical and Enzymatic Characterization of Leaves from Spanish Table Olive Cultivars
Source: Foods. 2022 Dec 1;11(23):3879. doi: 10.3390/foods11233879 (PMC9738326; doi:10.3390/foods11233879)

foods

MDPI

Table S1. Concentration of phenolic compounds, triterpenic acids and sugars (g/kg) in the olive leaves samples from 4 different orchards. Data are expressed as the mean values of duplicates. Standard deviation in parenthesis. "Others" is the sum of hydroxytyrosol 4-glucoside, hydroxytyrosol 1-glucoside, tyrosol, caffeic acid, verbascoside, ligustroside and luteolin 7-glucoside. Lowercase letters indicate significant differences among cultivars according to Duncan's multiple range test ($p < 0.05$).

	Phenolic compounds		Triterpenic acids		Sugars			
**Variety**	**Oleuropein**	**Others**	**Maslinic acid**	**Oleanolic acid**	**Sucrose**	**Glucose**	**Fructose**	**Mannitol**
Aloreña	62.43 (5.47)^{c}	6.03 (2.50)^{a}	2.51 (0.52)^{c}	14.71 (0.68)^{ab}	8.12 (4.22)^{a}	5.69 (1.93)^{ab}	3.69 (0.68)^{b}	22.79 (0.97)^{abc}
Cacereña	76.25 (9.06)^{ab}	4.69 (1.73)^{a}	2.87 (0.91)^{bc}	13.72 (3.67)^{b}	5.28 (1.45)^{b}	6.26 (2.95)^{ab}	3.66 (0.57)^{b}	22.10 (0.82)^{bcd}
Empeltre	72.50 (6.47)^{abc}	5.56 (1.05)^{a}	3.73 (0.64)^{a}	15.72 (1.84)^{ab}	4.32 (0.49)^{b}	5.25 (2.52)^{ab}	3.78 (0.57)^{b}	23.94 (2.07)^{ab}
Hojiblanca	65.70 (10.73)^{bc}	4.55 (1.47)^{a}	2.65 (0.60)^{c}	17.47 (2.54)^{a}	3.45 (1.46)^{b}	4.48 (2.15)^{b}	3.54 (0.43)^{b}	20.59 (1.98)^{cd}
Manzanilla	79.86 (13.06)^{a}	4.76 (2.05)^{a}	3.15 (0.49)^{abc}	16.77 (1.17)^{a}	4.31 (0.71)^{b}	6.19 (2.64)^{ab}	3.83 (0.94)^{b}	19.76 (3.53)^{d}
Verdial	71.23 (8.03)^{abc}	4.85 (0.69)^{a}	2.98 (0.65)^{abc}	14.75 (3.11)^{ab}	5.07 (2.00)^{b}	6.12 (3.25)^{ab}	3.90 (0.78)^{ab}	21.05 (2.20)^{cd}
Gordal	50.10 (11.07)^{d}	5.08 (1.26)^{a}	3.68 (1.07)^{ab}	17.26 (2.32)^{a}	4.03 (0.68)^{b}	4.60 (1.34)^{b}	3.50 (0.53)^{b}	24.67 (1.19)^{a}
Morona	63.97 (12.96)^{c}	3.45 (1.54)^{a}	2.86 (0.66)^{bc}	16.28 (2.18)^{ab}	5.46 (0.93)^{b}	8.20 (3.18)^{a}	4.60 (0.73)^{a}	23.61 (2.13)^{ab}

Figure S1. Polyphenol oxidase (A) and peroxidase (B) activities (U/mg protein) in olive leaves of Manzanilla, Gordal and Hojiblanca varieties collected from October to August during the 2020/2021 season. Data are means of duplicates. Vertical bars mean the standard deviation. Different letters indicate significant differences among harvesting times for each variety according to Duncan's multiple range test ($p < 0.05$).

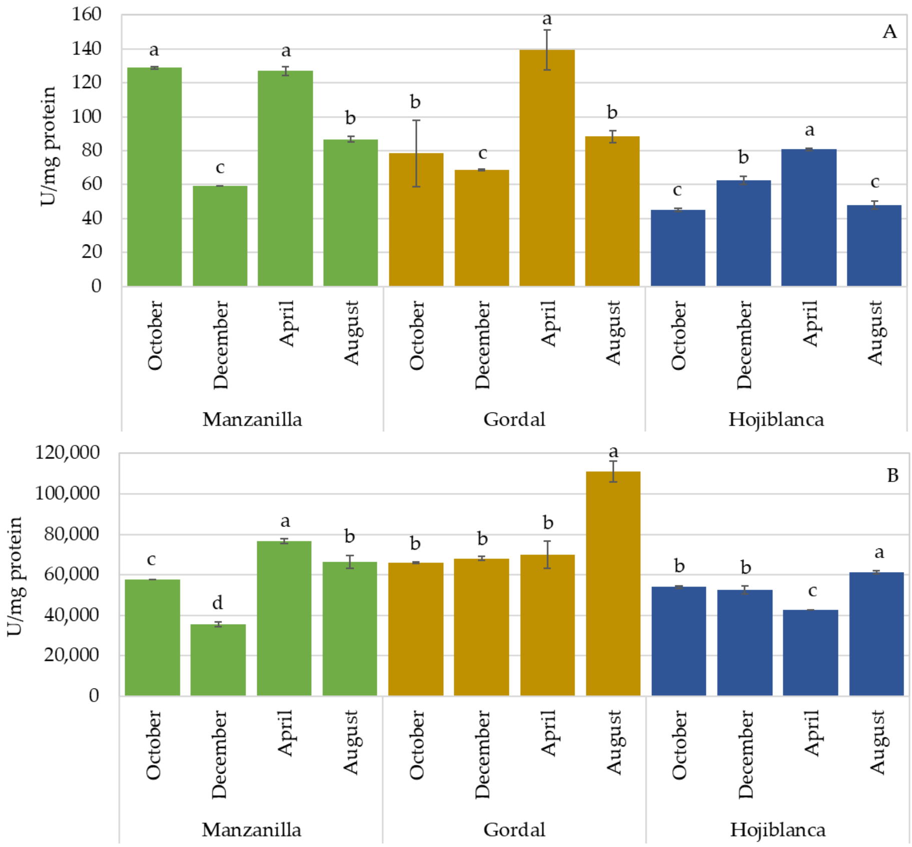

Supplement: Supplementary file 1 [file foods-11-03879-s001.zip › foods-1990869-supplementary.pdf]
